# Dental Surgery

**Published:** 1860-07

**Authors:** P. G. C. Hunt


### DENTAL SURGERY.

BY P. G. C. HUNT.

[A paper read before the Local Medical Society of Indianapolis, Indiana.]

DENTISTRY is said to be a speciality of medical science, and, though the profession is prone to regard (and perhaps often justly,) the assumption of special attention to, and skill in one branch of medical science, as savoring of the unclean thing, charlatinism, yet we are proud to say and believe that Dental Surgery and Mechanism is worthily taking a stand far in advance, of what in former years it occupied, and that too, with the full concurrence and entire approbation of the whole medical profession. The progress of the Dental art within the last half century has been truly astonishing. Then, how meagre its literature, how few its intelligent practitioners, and how comparatively unsatisfactory the best skill in the world—and how numerous were the pretenders to this liberal art, whose utter ignorance and dishonesty brought contempt upon themselves, and distrust upon the profession, and until comparatively recent date it is to be confessed, we imagine, that medical knowledge partook more of a theoretic or speculative tendency than at the present day.

Men of science felt inclined to allow Surgery and every thing mechanical connected with the healing art, to remain in the hands of Barber Surgeons, and gradually only did general surgery assume its present rank in the arcana of medical science. The history of science is a paralell case. Separate from it a knowledge of general and comparative anatomy, physiology, chemistry, and therapeutics, and what is there left? Why, not enough of vitality to keep it from relapsing into the shades from whence the last half century rescued it. A knowledge of medical science and its collateral teachings is the foundation upon which modern dentistry rests, and we can refer, with perhaps pardonable pride, to American Dental Literature, as the evidences of progress in theoretical and practical knowledge in this speciality, and the advance toward perfection in the mechanical department of the profession, is more rapid at this time, than at any former period, and the dentist who operates to-day precisely as he did ten years ago, is behind the times and fails to give his patient the benefit of much that has been learned and tested in that time.

Much that was formerly considered impossible in practical dentistry, is now a matter of every day operation, as for example, the restoration to health and usefulness of a tooth, the pulp of which is exposed by decay of bone, or even when after destruction of the nerve pulp suppurative inflammation has attacked the adjacent tissues, success in such an operation would scarcely have been hoped for a few years since, while at this day any patient who keeps pace with the advancement of the profession, will undertake without hesitation to perform such an operation, time and expense being considerations for the patient.

That there are important considerations for a general practioner, to be found in connection with those parts of the physical system, which pertain to dental education, is without doubt more or less known and acknowledged by the medical profession. But that the full measure of relation between the various conditions of disease, loss of natural organs, enervating salivary secretions, impure exhalations issuing from the mouth contaminating the air we breathe, is not fully understood can not admit of a doubt, all of which and more, it is the special province of dentists to investigate and endeavor to remedy. The full relations between these conditions and other parts of the animal economy, in relation to general diagnosis curative and sanative measures are not generally accorded by physicians. In endowing man with a complex structure and arranging the multitude of glands, organs and tissues, intimately connected with each other; the lips, the tongue, the salivary glands, and especially the teeth, reasoning *a priori*, we should suppose are of such importance to a healthful state of the animal system that their loss or an impairment of their functions, would result in a disturbance of those other organs, with which they sustain the most intimate relations. Nor will it be found that such reasoning is fallacious. Any one who has had occasion to observe the general health of a person during a series of years, which shall include a period extending from a time when the teeth are healthful, intact, and performing fully the duty of mastication through the various gradations of disease, decay and loss, will inevitably discover dependent impairments of the digestive functions. Solid food or that which is possessed of moderate adhesion of its particles, no longer submitted to the grinding of the dental organs, and a thorough admixture with salivary secretions is passed into the stomach, unfitted for the ready action of that organ. Such an occurrence repeated daily, almost hourly, wearies out the ability of the digestive apparatus, and though the wonderful adaptability of the animal organism is sufficient to withstand for a time, this unnatural condition, its continual recurrence finally produces the most deplorable results. The external indications of which are irritability of temper, sallow complexion, pinched expression of countenance, and every indication of a confirmed dyspeptic, not to speak of a loss of the natural contour of the face and inability to articulate with distinctness.

Every general practitioner will recognize the sequence which we have described, yet not every one, it is to be apprehended, if consulted by a patient suffering under those circumstances, would recognize the important aid to be derived from the skillful services of an intelligent and conscientious dentist. But too frequently in such cases will the general practitioner after ascertaining debility and vitiated action in the digestive functions, substitute these for primary indications and found a treatment, on a partial diagnosis of the case. The insalivation of the food is strictly a mechanical operation, its trituration comingulation, with the secretions and its preparation into a bolus, its deglutition are all purely of mechanical nature, and a loss of those implements used in any of these processes, of course renders such process defective or impossible. As well might the miller expect to produce a double extra quality of flour, his neither millstone being missing, as to expect a mouth deprived of *grinders*, to prepare proper digestible material for the stomach. Now one province of the dental art is to supply a substitute for the loss of natural parts, and just so far as this can be done with success, should it be estimated, and receive the meed of favor which is its due.

Foreigners say of the American people, that we spend too short a time at the table and they ridicule with great apparent zest, the Amcrican method of "bolting food," and they argue with seriousness that mastication can not be sufficiently performed in the hurried manner in which Americans swallow food. Is there not reason in their more leisurely manner of satisfying the appetite? Evidently more perfect preparation of food can be had by eating with slowness.

In ruminating animals we find that perhaps one third of the twenty-four hours is spent in masticating the food, in strictly carniverous tribes, food is generally taken from necessity at considerable intervals, and instead of a masticatory process long protracted, the food is cut into pieces adapted to their purpose, having teeth not suited to comminuting or grinding their food. Man who occupies with respect to teeth and digestive system, an intermediate position is possessed of dental organs indicating that though he is not adapted to chew the cud like the former, neither should he be content, like the latter, to cut it into morsels fitted for the largest capacity of the Æsophegas. Reason, experience and observation, alike concur in pointing out the propriety of a thorough mastication of every solid edible, and those possessing imperfect grinders should occupy a time longer if necessary, or their food should be of such a nature as to require less trituration to reduce it to a proper consistency.

In civilized societies the teeth are among the first organs of the body to exhibit the deleterious effect of artificial and unnatural methods of living, and this may be observed both in their formation, which in the period of development, ill health, lack of appropriate nourishment may render defective. And in maturity, when though they may be normal in structure, they yield to the powerful solvents which bad digestion may produce, or bad taste select as condiments. When during the period of second dentition the health of a child is imperfect, observation will warrant us in predicting with a degree of accuracy that the teeth will be abnormal in their constituents, a deficiency in their gelatinous elements is mostly observable. They possess a whiteness of color, which is unnatural, they are friable and imperfectly protected by enamel, and not unfrequently their position and arrangement is irregular, and also we think that we have observed this connection between the development of teeth and the general system, that large perfect teeth are associated with large healthful bodily development, and *vice versa.* And as teeth are formed and their size determined before the completion of development of other portions of the system, does it not sometimes happen that the physical growth of an individual may be prematurely brought to a stand, and he possess teeth suited for a larger stature? We conceive we have observed this to obtain in many instances, we do not suppose that in a physiological condition the teeth should invariably bear a precise ratio in size to physical development, but we think that an approximation can readily be discovered by attention, much more so than in the hand or foot or other organs of the body. Without doubt it can be said, that the general practitioner does not lose sight of diseases of the mouth, but the dentist who makes these diseases, his speciality should be awarded that position by the medical profession that his acquirements as a specialist seem to demand.

Eminent minds have often admitted, that a single department of science is sufficiently comprehensive to occupy the attention for the entire life of one man. With the dentist in full practice, it is a matter of every day occurrence to see mouths so replete with salivary calculus and accumulated filth, as to leave no doubt in the mind of any one, who reasons from cause to effect, that to this cause, in many cases, might with truth be referred disturbances, more or less serious in the alementary canal and lungs. Again that most distressing disease, facial neuralgia, which is considered unmanagable and eccentric, in a large proportion of cases by a careful diagnosis will be found to depend upon the diseased pulp of some offending tooth. In whatever light dentistry may be viewed, it will be evident to an unprejudiced mind, that it has claims to be admitted to the favorable consideration of the medical profession, whether as a collateral branch of the healing art, or as means of relieving human suffering and contributing to the comfort and happiness of mankind.

